# Exploring the implementation of an electronic record into a maternity unit: a qualitative study using Normalisation Process Theory

**DOI:** 10.1186/s12911-016-0406-0

**Published:** 2017-01-07

**Authors:** Arabella Scantlebury, Laura Sheard, Ian Watt, Paul Cairns, John Wright, Joy Adamson

**Affiliations:** 1 York Trials Unit, Department of Health Sciences, University of York, York, YO10 5DD UK; 2 Bradford Institute for Health Research, Bradford Royal Infirmary, Duckworth Lane, Bradford, BD9 6RJ UK; 3 Department of Health Sciences, Seebohm Rowntree Building, University of York, York, Yo10 5DD UK; 4 Department of Computer Science, University of York, York, Yo10 5GH UK; 5 Institute of Health and Society, Newcastle University, Newcastle Upon-Tyne, NEZ 4AX UK

**Keywords:** Normalisation Process Theory, Electronic records, Maternity, Benefits, Implementation, Patient safety

## Abstract

**Background:**

To explore the benefits, barriers and disadvantages of implementing an electronic record system (ERS). The extent that the system has become ‘normalised’ into routine practice was also explored.

**Methods:**

Qualitative semi-structured interviews were conducted with 19 members of NHS staff who represented a variety of staff groups (doctors, midwives of different grades, health care assistants) and wards within a maternity unit at a NHS teaching hospital. Interviews were conducted during the first year of the phased implementation of ERS and were analysed thematically. The four mechanisms of Normalisation Process Theory (NPT) (coherence, cognitive participation, collective action and reflexive monitoring) were adapted for use within the study and provided a theoretical framework to interpret the study’s findings.

**Results:**

Coherence (participants’ understanding of why the ERS has been implemented) was mixed – whilst those involved in ERS implementation anticipated advantages such as improved access to information; the majority were unclear why the ERS was introduced. Participants’ willingness to engage with and invest time into the ERS (cognitive participation) depended on the amount of training and support they received and their willingness to change from paper to electronic records. Collective action (the extent the ERS was used) may be influenced by whether participants perceived there to be benefits associated with the system. Whilst some individuals reported benefits such as improved legibility of records, others felt benefits were yet to emerge. The parallel use of paper and the lack of integration of electronic systems within and between the trust and other healthcare organisations hindered ERS use. When appraising the ERS (reflexive monitoring) participants perceived the system to negatively impact the patient-clinician relationship, time and patient safety.

**Conclusions:**

Despite expectations that the ERS would have a number of advantages, its implementation was perceived to have a range of disadvantages and only a limited number of ‘clinical benefits’. The study highlights the complexity of implementing electronic systems and the associated longevity before they can become ‘embedded’ into routine practice. Through the identification of barriers to the employment of electronic systems this process could be streamlined with the avoidance of any potential detriment to clinical services.

**Electronic supplementary material:**

The online version of this article (doi:10.1186/s12911-016-0406-0) contains supplementary material, which is available to authorized users.

## Background

E-health - the use of information technology for health - is increasingly viewed as a tool for transforming the healthcare industry and a mechanism for improving the efficiency, quality and safety of care provided [1, 2]. In recognition of the growing international interest in e-health, since 2010 the Global Observatory for e-Health has maintained an online repository for e-Health related national policies and strategies for World Health Organisation member states [3].

A key focus of global e-Health policy is for healthcare organisations to implement electronic records [3]. However, globally, progress in implementing these systems has been varied; despite Denmark, Canada, Australia and England all proposing national strategies to implement electronic records and promoting information sharing between and across healthcare organisations over the last decade [3]. In contrast, significant progress has been made in the U.S, where the majority of hospitals have implemented electronic records and are now transitioning from one type to another [4]; the success of which is partly due to the American Government, who committed $34billion to incentivise health professionals to use certified electronic records in a meaningful way [1].

A recent policy paper – ‘Personalised health and care 2020’ [2] - proposed an ambitious target for all NHS hospitals to be ‘paperless’ by 2020. However, this goal has been revised following recommendations from the Wachter Review, which assessed the progress of the NHS in relation to its digital vision [5]. To support the new ambition for a paperless NHS by 2023, the Health Secretary has committed £4.2 billion over the next 5 years [6]; which at a time when a funding gap of £30 billion has been predicted for the NHS [7], highlights the government’s urgency and belief that NHS IT will reap significant rewards.

As electronic records are high on the health agenda for many countries, evidence of how best these systems can be implemented is of international importance. Empirical evidence that explores the benefits, barriers and disadvantages of implementing electronic records has recently been summarised in a systematic review [8] of 22 studies, which identified technical issues alongside financial and time constraints as the most frequently reported barriers associated with electronic record implementation. However, the current evidence base is dominated by studies from the US and given the major differences in the social, political and economic foundations of their healthcare system, it is important to explore whether these issues are relevant in other contexts.

This study aimed to explore the benefits, barriers and disadvantages of implementing an electronic record into a specific UK NHS context – a maternity unit. This particular setting was selected as record keeping in maternity care differs to other specialties as women are responsible for their paper records, with their care documented on the paper record at each visit to community or hospital-based healthcare [9]. Paper records have been widely used in maternity care since the co-operation card was introduced in the UK in 1956, which made paper records a successful and integral tool for maternity shared care [10]. A recent systematic review noted that clinicians working within the GP-maternity shared care environment had positive perceptions of the paper record; however the response to electronic records was more mixed [9]. Given the enduring positivity toward paper records and observed reticence toward electronic systems amongst clinicians, by exploring in-depth the process of implementation and the extent to which the electronic health record had become embedded into routine practice, important insights could be uncovered.

## Methods

### Study design

A qualitative semi-structured interview study was adopted to explore participants’ perceptions and experiences of the benefits, barriers and disadvantages of implementing an electronic record in a maternity unit. Interviews were conducted face-to-face with health practitioners working within the maternity unit.

### The study site and electronic record system

This study explores the implementation of an electronic record into a single case site-a maternity unit within a NHS teaching hospital in the North of England. The maternity unit, which offers services to approximately 6000 women and families annually is comprised of the following wards: antenatal day unit, birth centre, labour wards, antenatal/postnatal wards and the maternity assessment centre. The study provides an in-depth exploration of an electronic record implementation at a large inner-city NHS hospital and so was considered representative of other UK trusts. It was anticipated that a broad range of factors affecting implementation, applicable to other NHS trusts would be identified.

In 2007, the maternity unit under study implemented an electronic system as a result of the Department of Health informatics directorate that was expected to create a paperless environment. However, the initial system did not reach its full potential and so the electronic record under study here was introduced as a replacement. This new system was introduced in a phased manner and at the time of the qualitative interviews was being used in the community, labour ward and for antenatal care. During the study period, the system was also introduced into post-natal care. Despite implementation in and across the maternity unit, the system was not considered to be at full capacity and the unit were regarded as having a mixed (paper and electronic) record. For example, in labour ward intrapartum care was documented on the paper record, with delivery summaries recorded on paper before being entered onto the electronic system. Additionally, the use of the electronic record system varied throughout the maternity unit as different specialties and staff groups (doctors, midwives, health care assistants) have different requirements for the system. This is reflected in the range of healthcare activities for which the system was being used throughout the maternity unit which included: research, discharge notifications to GPs, clinical observations, antenatal assessments, alerts for risk factors and allergies, care delivery and operative documentation. To protect the trust and suppliers anonymity, the electronic record system is referred to as the ‘ERS’ throughout this paper.

### Theoretical approach

This study draws upon Normalisation Process Theory (NPT) [11, 12], a theory that is used to explain the factors that promote or inhibit healthcare technologies from being embedded into practice [12, 13]. The theory states that the work of implementing a technology is achieved through ‘energising’ four mechanisms: coherence, collective action, cognitive participation and reflexive monitoring [11]. The four main mechanisms of NPT were considered a useful way of identifying factors affecting the ERS’ implementation and for determining the extent that the system has been embedded into routine practice. NPT was used to inform the interview schedule and provided a theoretical framework to interpret the study’s findings. Murray et al. [14] use a number of examples to demonstrate how researchers can apply NPT to the design, evaluation and implementation of studies in healthcare. The work of Murray et al. [14] was used as a guide to create the following broad working definitions, so that NPT could be adapted for use within this study and ensured that the interview schedule included questions relating to all four mechanisms of NPT:

- ***Coherence:*** Do staff have an understanding of why the system has been implemented?
- ***Cognitive participation:*** Are staff engaged and committed to using the system and what are the factors that promote and/or inhibit this commitment?
- ***Collective action:*** Are participants using the system and what are the factors that promote and/or inhibit them from using the system?
- ***Reflexive monitoring:*** Have staff appraised the system and its impact on practice?

### Sampling and recruitment

The ERS was introduced progressively throughout the maternity unit and so interviews were conducted between April and November (2014) during the first year of implementation. The phased approach to implementation meant that at the time of interview, the length of time participants had been using the ERS varied. This was taken into account in the sampling strategy - a purposive sampling frame was used to ensure that interviewees represented a variety of staff groups, grades and wards as it was anticipated that staff would have different usage and experience of the ERS depending on their roles and responsibilities. In addition to different grades of midwife (e.g. NHS band 6, NHS band 7), doctors from Senior House Officer to Consultant were recruited. Although Health Care Assistants (HCA) use of the ERS was limited, this professional group were included as it was anticipated that their practice would be altered by other clinicians’ use of the system.

Clinical staff involved in supporting the implementation of the ERS were also recruited as it was expected that their perceptions and experiences of the ERS may differ from those not actively involved in implementation. These staff operated through two main groupings – “the support team” and “super users”. The support team were clinicians responsible for championing and assisting their colleagues in using the system and so were directly involved in ERS implementation. Super-users were predominately medical consultants at the trust who had received extra ERS training. These individuals assisted clinical colleagues, during their shifts and so provided additional assistance when the support team were unavailable (outside of weekday office hours). Participants were recruited via telephone, email and a junior doctors WhatsApp group. Participants were sampled until a range of specialities and professions within the maternity unit were represented and no new themes emerged.

### Participants

19 participants consented and were interviewed. Participant characteristics are displayed in Table 1. Interviewees included 11 midwives (grades 5–7), 7 doctors (Senior House Officer to Consultant) and 1 HCA, representing a range of wards throughout the maternity unit including: maternity assessment centre, community, birth centre, labour and the antenatal day unit. Of this sample, 4 interviewees were involved in the ERS implementation, as members of the support team or super-users.

**Table 1:** Participant characteristics

Participant ID	Profession	Years experience	Years at trust
013009	Health Care Assistant	6	6
023010	Midwife	7	7
030211	Midwife	11	9
042202	Doctor (consultant)	7	7
051610	Midwife	14	14
062712	Midwife	28	34
070202	Doctor (consultant)	11	11
081203	Midwife	14	10
091203	Midwife	3	3
101310	Midwife	23	28
111609	Doctor (registrar)	7	8 months
122309	Midwife	15	15
133002	Midwife	7	7
140308	Midwife	25	25
150308	Midwife	23	23
161111	Doctor (registrar)		1
170210	Doctor (registrar)	5	7 months
180703	Doctor (consultant)	16	
191812	Doctor (consultant)	21	21

### Interview design and content

Interviews were conducted face-to-face, were semi-structured and lasted between 17 and 42 min. A topic guide (Additional file 1) provided the framework for the semi-structured interviews and was informed by relevant research and NPT [12, 14]. The four mechanisms of NPT were used to shape the questions within the topic guide. Interviewees were asked about their perceptions and experiences of the benefits, barriers and disadvantages of the ERS, why they felt it had been introduced, the extent that paper records were still used and the impact of ERS on practice.

### Reflexivity

A reflexive approach was taken to data collection and analysis. AS was responsible for data collection and analysis and undertook this work as part of her doctoral thesis which explored the implementation of electronic records into NHS secondary care organisations. AS had no involvement in the ERS implementation and prior to her PhD had no experience of NHS IT. Other members of the research team had an academic research background (JA, IW, PC, LS); JW and IW also had a clinical background. None of the research team were involved in the ERS implementation or worked in the maternity unit. The research team may therefore be considered to be in a neutral position relating to any prior expectations to the study or the ERS' implementation.

### Analysis

Interviews were audio-recorded and transcribed verbatim with each participant assigned a unique ID code for anonymity. Reflexive notes [15] were taken after each interview with personal and methodological issues and challenges noted. Interviews were analysed using the stages of thematic analysis as outlined by Braun and Clarke [16]: familiarisation, code, theme development and data reporting [16]. Theme and sub-theme development was largely deductive, using a-priori codes dictated by interview questions (e.g. benefits participants expected prior to ERS implementation). Following, the initial thematic analysis a secondary analysis was conducted to explore the extent to which the themes developed in the initial analysis mapped onto the four core mechanisms of NPT. By doing so we were able to examine the extent which the ERS was thought to be embedded into routine practice. AS conducted the analysis alongside regular discussions with JA and LS in order to scrutinise the robustness of the theme development and mapping the themes onto the concepts of NPT. This was particularly important, given the dynamic and inter-related nature of the four components of NPT, which meant that a number of themes could be placed under multiple components. For example, whether an individual understands the reasons for the system being implemented (coherence) is thought to affect how they engage with (cognitive participation) and use the system (collective action). NPT also suggests that these three mechanisms relate to how individuals appraise the system (reflexive monitoring).

## Results

Whilst the four mechanisms of NPT provided a framework for structuring the findings, for interpretation purposes, the inter-related nature of these should be considered.

### Coherence – participant understanding of why ERS has been implemented

Participants were divided regarding their understanding of why the ERS had been implemented. Amongst those with responsibility for facilitating implementation and assisting colleagues with ERS, coherence was strong - coinciding with the official perspective of the institution as enabling the organisation to move towards a paperless environment. These participants attributed ERS implementation to the need for improved: accessibility and availability of records, efficiency, research and communication with other health and care organisations. They also had strong expectations that the system would bring benefits and have a positive impact on practice. For example, some participants reported that they anticipated it would remove risks associated with patients losing and forgetting paper notes, improve clinical audits and facilitate enhanced access to patient information, as staff assumed it would be integrated within and between healthcare organisations. A reciprocal relationship existed between participants’ ‘coherence’ regarding ERS implementation and whether they thought the system would lead to benefits prior to its implementation.

*Doctor (consultant) 070202: There were lots of negatives with hand written notes they were often not contemporaneous bits of paper go missing so…I mentioned wanting something robust to stand up in court with but paper notes may not provide that…either so…you know hopefully it was going to fill some gaps left by paper notes and hopefully allow better communication with other healthcare providers.*

This was in contrast to those (the majority) who had no involvement in ERS implementation who felt that they had not been informed as to why the system was introduced, therefore, did not have the anticipated benefits, causing some clinicians to feel as though the new system had been enforced upon them without explanation.

*Specialist Senior Midwife 051602: ‘well somebody likes it so that’s why we’re doing it’ that’s been said and ‘even If it doesn’t work we’ve got no choice’ has also been said.*

### Cognitive participation – staff engagement and commitment to ERS

If a technology is to be embedded into routine practice clinicians need to be prepared to invest their time and be engaged in its application (cognitive participation) [14]. This is dependent on certain factors that ‘promote or inhibit’ individuals use of the technology, which are discussed below.

#### Training and support

The trust attempted to ensure participant’s continued commitment to engaging with and using the ERS by providing additional resources such as extra training, ‘lessons learned’ emails and electronic guidance for complex tasks; clearly if staff are expected to use the system, they need to understand how to use it. However, participants criticised the delivery (too simplistic, dogmatic), varied content (some staff only received basics of ERS) and timing (too far in advance of or after implementation) of training. The amount of training received also varied, with some participants receiving none, a single 30 min or whole day training sessions. A senior midwife attributed this variation to different roles of staff requiring different uses of the ERS, however participants cited issues with staff being able to ‘fit in’ training amongst busy work schedules and shift patterns:

*Midwife (Birth Centre) 091203: every so often they’d put a few days in but you’ve got midwives that work permanent nights so how do you catch them?.*

In addition to formal training, throughout implementation, a support team that consisted of a group of seconded members of clinical staff were responsible for helping staff to use the system. Despite criticising the support team’s availability (Weekday office hours only), participants praised their assistance during early implementation; particularly for those with poor computer literacy. The support team were also responsible for rectifying data entry errors on the system made by staff, who only had the capacity to input, and so could not edit information within the ERS. A group of clinicians that received extra training on the system who were considered ‘super-users’ were available when they were on duty and helped clinical colleagues rectify errors made on the system:

*Midwife (Birth Centre) 081203: some of the more senior staff, I think they were called super-users they got additional training, so that was helpful in the unsocial hours, so obviously on a night shift, or on bank holidays, or weekends when the team weren’t there they could…problem shoot.*

#### Barriers to engaging with the system

Participants perceived there to be a reluctance to accept the ERS and the change associated with its implementation among staff within the maternity unit, which negatively impacted on their engagement with and willingness to invest their time into the ERS. Despite an understanding among many that there were positive reasons for introducing the ERS, the historical use of paper records (and the positive view of these) made staff hesitant about the prospect of a paperless environment.

*Midwife (labour ward and maternity assessment centre) 133002: I have a specific way of writing it and I have written it that way for an awfully long time and when I go to type it and writing a very small box although I can put as much in there as I want it doesn’t flow as easily…things like that and…it feels a little bit disjointed whether that will improve the more we do it but I am worried that there will be an issue.*

Secondly, participants felt that the maternity unit had already been subject to vast amounts of ‘top-down’ policy change (from local and wider NHS initiatives) relating to increased data collection and audit requirements for maternity services. Additionally, participants who had been working at the trust for a number of years were affected by the implementation of the former (failed) electronic record system and did not distinguish between the two systems. These individuals had expected to be using an ERS within a paperless environment 7 years ago and so viewed the implementation as slow and with scepticism:

*Midwife (Maternity Assessment Centre) 062712: the way it has been rolled out with them saying it will be rolled out in six months and we are now 7 years down the line it is probably going to be…I will be retired by the time it comes in (235–237).*

### Collective action – ERS usage by participants

In addition to individuals understanding why the ERS was introduced (coherence), or willingness to engage with and invest time into the system (cognitive participation) the following benefits and barriers may have promoted or inhibited the extent that the ERS was used.

#### Realised benefits

Although some participants reported that they were yet to see clinical benefits from the ERS, others felt that benefits had started to emerge. As expected, in contrast to paper records, the ERS was perceived to have enabled more reliable clinical audits to be conducted. Participants also reported that as more reliable information relating to the case mix of the maternity unit and work patterns is collected, financial benefits are occurring as the hospital is now able to charge commissioners (Clinical Commissioning Groups) more accurately for the care it provides:

*Doctor (Consultant) 180703: we were struggling to charge the correct tariffs and we could see that a computer system like this was going to make it easier for us to charge the correct tariffs from the CCGs for the pregnant women and that has proven correct (52–55).*

Participants cited a number of clinical benefits, which were largely associated with staff having access to patient records 24 h a day and records no longer being the patients' responsibility. Participants provided a number of examples, where this has been beneficial to the safety and quality of care provided. For instance: checking the importance or reason for visits prior to appointments; alerting community midwives in the event of patients failing to attend appointments; mitigating risks associated with patients forgetting or losing their records and accessing records in emergencies. Further benefits of the ERS in comparison to paper records included: simple data entry methods (e.g. tick boxes), prompts for additional information during alternative care (e.g. water births), improved communication with GPs who can now receive electronic notifications when patients are discharged or prescribed medication and increased legibility and conciseness of records. Clinicians also acknowledged that they no longer have to write the same information into numerous forms as the ERS populates relevant sections of the record. These findings suggest that participants had some positive experiences of, or awareness of colleagues having realised benefits from using the ERS since its implementation, and this in turn would be expected to positively influence their continuing engagement with (cognitive participation) and use of the ERS (collective action):

*Midwife (Antenatal clinic and day unit) 150308*: *I’m not having to try and read illegible handwriting now because that’s always been a major barrier with providing care (143–144).*

#### Barriers to using the system

The ERS was not ‘fully’ implemented during the period when the interviews took place and so not all aspects of care were inputted onto the ERS e.g. anaesthetic alerts; with paper still used in these situations. Paper was also used to communicate with other departments due to the ERS not being integrated with other electronic departmental systems within the trust. The mix of paper and electronic media and lack of integration between departmental systems was perceived to have raised the risk that clinical information may be missed. Additionally, the ERS could not communicate or share information with other healthcare organisations, with the procedures for granting other organisations access either unknown or considered too complex. This was considered an additional risk of the ERS as previously, unless women lost or forgot their paper records, they would have had them on their person when attending other healthcare organisations. One participant described the implications of these issues for participants who relocate for safe guarding issues:

*Midwife (Birth Centre) 081203: women who haven’t booked with a midwife who may be moved from a different area because they are trying to go under the radar, they might have safe guarding concerns, they might be frightened that their baby is going to be taken away from them and they deliver at other trusts and that’s a way to try and escape that and we don’t have access to that persons records if they come from somewhere where they don’t have our system (177–181).*

The staged approach to implementing the ERS meant that the extent that paper was used throughout the maternity unit varied, with some wards described as paperless whilst others were reliant on paper or both. Participants using both paper and electronic records expressed their frustration at the additional time it was taking them to ‘do everything twice’. Participants also raised concerns that important information may be being missed or not documented adequately in either record system. A variety of reasons for this were provided including: perceptions that some staff still see the paper record as the primary record, greater detail being entered into paper notes than on the ERS, staff not being aware and/or checking both sets of notes and insufficient time to document in both records:

*Doctor (Registrar) 161111: I know that the system team they are stressing on the point that everything should be on the system, however for one reason or another I don’t know whether the systems down or whatever, some patients they still do have handheld notes or they have some of the documentation of their history on the paper work and other things on the system (107–109).*

In addition, some staff perceived the ERS to increase the potential for inputting errors, particularly following system upgrades or when new members of staff (e.g. junior doctors) that were not used to the ERS joined the wards; which also contributed to their unwillingness to use the system:

*Doctor (consultant) 191812: Our junior medical staff change, anything from every four to every 12 months and when our new staff come then it takes them a while to get used to it. So introducing people to the system takes longer and as I say we just upgraded it to change and so all of us go back a step in terms of learning (180–184).*

### Reflexive monitoring – staff appraisal of the ERS

Throughout the interviews, participants appraised the system by identifying a number of additional factors that have promoted (future benefits) and inhibited (disadvantages) their use of the system.

#### Disadvantages

Some participants perceived it to be more time consuming to enter information onto the ERS compared with paper records. Participants also explained how technical issues such as the system crashing and the time required to log into the ERS for each patient was lengthening appointments and discharge. Whilst some participants who had been using the ERS for longer did explain that the ERS was becoming quicker to use, many felt that the trust were underestimating the added time pressures associated with the ERS:

*Midwife (Maternity Assessment Centre) 062712 I can’t see it [entering information electronically] being feasible when it’s very busy for me to physically be able to do it and then I’ll have concerns over my record keeping (202–203).*

A minority of participants anticipated that although they expected the ERS to negatively affect their interaction with patients, ‘they made a concerted effort’ and had successfully avoided this. For participants who felt that the system had a detrimental effect on their relationship with patients, this was attributed to staff being required to leave the bedside to access the computer. Participants also described how because they had to physically turn away from the patient and concentrate more when using the computer, they felt they were not giving patients enough attention. However, of those that reported a negative impact, a proportion felt that they are now spending as much time with patients as they did when using the paper records and suggested that the detrimental impact on their interactions may be constrained to early implementation. Any potential detrimental impact of the ERS and interaction with patients was also perceived to have consequences for patient safety:

*Doctor (Consultant) 070202: I don’t have a midwife in the clinic with me anymore because she has to log in separately and put her information in and there seeing patients separate to us. So particularly when there is a complex psycho-social case, maybe domestic violence, maybe extreme poverty, drug issues whatever, previously you would see them together, so you would establish a bit of a rapport a relationship with the patient and one of you would pick up on some things the other will pick up on others. You need to approach those cases subtly now they’ll go to a midwife who just does the blood pressure and the way make sure they’ve got the right leaflets and then they come along to me for the medical consultation….and I won’t be aware of what’s gone on in the midwives room (123–128).*

#### Anticipated benefits

In light of the limited benefits and various barriers and disadvantages experienced since the ERS was implemented, it may be that as well as being a requirement to undertake their job, staff continue to use the system as they expect benefits to emerge in the future. A number of participants reported expectations that the ERS, once fully implemented, will enable all patient information to be stored in one place; something which is predicted to be of benefit in emergency situations as the ERS will alert staff to allergies and risk factors. Once the ERS is integrated within and across healthcare organisations, participants also expected quicker referral times as they will no longer have to wait for letters. Additional anticipated benefits included improvements to: patient flow, research, audit, performance and planning, record security and accuracy and fewer missing records:

*Doctor (Consultant) 042202: in an emergency situation as soon as I know name and date of birth or something like that, if I open that I know about…yes this women had a road traffic accident and such and such she had a blood transfusion such and such and she got allergy to penicillin and she is now 28 weeks pregnant. If the women is not in a state to talk to me that is one I’m expecting… so that has to be able to give me that complex background (127–134).*

## Discussion

This study explored the perceptions and experiences relating to the implementation of an ERS amongst healthcare professionals working in a NHS maternity unit- in terms of the benefits, barriers and disadvantages. NPT has characterised a range of factors that have helped healthcare professionals to; understand the purpose of (coherence), engage with (cognitive participation), use (collective action) and appraise (reflexive monitoring) an ERS. The fact that participants in our study still used paper and in some cases viewed the paper record as the primary record, suggests it is taking a long time for the ERS to become an established part of routine practice. The extent of ‘normalisation’ reported here, was largely a result of human (e.g. computer literacy), organisational and contextual factors (e.g. the previous failed implementation of an ERS) and the phased approach to implementation; which resulted in the parallel use of paper and slow implementation of the ERS throughout the maternity unit.

Our study has identified a number of barriers to implementing an ERS within a maternity unit. This is consistent with a recent systematic review of systematic reviews that explored factors influencing the implementation of e-health [17]. The review concluded that issues around e-health implementation are both multi-level and complex, as no single factor was identified as a key barrier or facilitator [17]. Therefore, organisations may wish to consider the specific context into which an ERS or e-health technology is being implemented, in order to understand which factors may affect their implementation. For example, in our study whilst a range of different barriers to implementation were reported, a large proportion of these were a result of the phased approach to implementation and the parallel use of paper within the maternity unit.

Our study also highlighted that some individuals were hesitant about the prospect of working within a paperless environment, with some individuals preferring to use paper for certain tasks. This is consistent with a systematic review of paper and electronic health records within a maternity shared-care environment that reported that whilst women and healthcare professionals generally spoke positively about paper records, they held mixed opinions towards electronic maternity records [9]. Additionally, and in support of our study’s findings, whilst health professionals largely accepted the electronic record and felt it increased the reliability and legibility of information they found it time consuming and reported issues with accessing computers [9].

Although there is international pressure for hospitals to implement electronic records, based on the belief that these systems will transform the quality and safety of healthcare [1, 2], there is little empirical evidence to support this. Existing literature on the benefits of electronic records is largely U.S or primary care based [18–34] and reports ‘potential’ rather than actual benefits; with a large proportion of this evidence reporting the potential for electronic records to improve patient safety [19, 21, 23, 27–30]. This is consistent with findings from our study, as even individuals that were yet to see any actual benefits of the ERS believed that the ERS would lead to benefits in the future (e.g. through alerting to allergies and risk factors in emergencies). In accordance with existing literature, evidence of actual benefits reported in our study were limited to a number of ‘clinically orientated’ benefits surrounding improved information availability, accessibility and legibility [19, 23, 31–34]. This study has also added to an emerging but limited UK evidence base that has reported potential negative impacts of electronic records on the doctor-patient relationship [35] and patient safety in secondary care [36–38]. If NHS trusts and policymakers are to justify their political and financial commitment to implementing electronic records, further UK evidence is required to explore clinical benefits in more depth and identify the ‘quantifiable benefits’ (patient safety and efficiency) that are the main drivers of NHS IT policy.

This study was conducted with NHS staff from a single maternity unit within a large inner-city NHS hospital. Given the pressure on hospitals to implement electronic records globally, it is anticipated that some of the benefits, barriers and disadvantages are transferable and will provide useful insights to other healthcare organisations implementing similar systems. The study also explored the implementation of a single electronic system, with a number of the barriers and disadvantages being the result of design and implementation issues of that specific system. Nevertheless it is unlikely, that barriers such as technical issues are only a challenge with this system, as supported by technical issues being a frequently cited barrier to electronic record implementation within the literature [25, 26, 31, 34]. The phased-approach to implementing the system meant that interviews were conducted with participants who were experiencing different stages of implementation, with some of the issues identified a direct result of the phased implementation and the subsequent dual use of paper and electronic records. For example, the additional time to input information into both records and the potential for clinical information to be missed if health professionals do not check both records.

### Recommendations

To ensure effective change management when implementing electronic records healthcare organisations should ensure that staff’s expectations are managed. Transparency surrounding the reasons for introducing these systems, the timescale when benefits are expected to emerge and what barriers and disadvantages staff may experience; particularly during initial implementation is recommended. The variation in staff’s computer literacy also warrants consideration and is likely to be an issue for healthcare organisations implementing technology globally. To overcome this, training should not be delivered too far in advance of systems being implemented and should be undertaken by all members of staff, taking into account staff availability and shift patterns.

Although healthcare organisations need to identify quantifiable benefits (e.g. efficiency savings) to justify financial and political commitments to electronic records, it is important that the more clinically orientated benefits identified here (e.g. improved information availability), are established; particularly if staff are to engage with and accept new technology.

A number of the issues reported in this study were a result of the phased approach to implementing the ERS. This provides some evidence of negative consequences associated with implementing electronic records using a phased approach. However, evidence regarding the pros and cons of different approaches to implementing electronic records is limited [39]. Given the limited guidance available [40] and the lack of agreed ‘best method for implementing electronic records, further research is required.

In light of the global investment into electronic records and the complexity associated with their implementation, further research is essential if the factors affecting implementation and benefits and disadvantages of these systems during initial implementation and the longer term are to be understood. This is of particular importance when considering that NHS trusts require evidence to formulate their business cases and benefits realisation plans that support bids for and show returns of investment respectively. However, the heterogeneity of electronic record implementation (different hospitals, approaches, systems) means Randomised Controlled Trials in this area are difficult. Future research will need to adopt a range of observational and qualitative methods to build on existing evidence. Research that identifies the benefits of introducing electronic records into the NHS, through longitudinal and/or before and after studies to identify the benefits of these systems throughout implementation and in the longer term should be prioritised.

## Conclusions

This is the first study to qualitatively explore clinicians’ perceptions and experiences of a maternity system’s implementation into an NHS trust. The study expands on the limited UK evidence and adds to international evidence surrounding electronic records, by using NPT as a framework to identify the benefits, barriers and disadvantages of implementing an ERS during the early stages of implementation. The study has added to a body of mainly US literature, which has identified potential disadvantages of electronic systems. Given the complexity of implementation and the pressure on healthcare organisations to become paperless, further research is required.

## Additional file

Additional file 1: Topic guide for interviews with NHS Staff: topic guide used for the qualitative interviews with study participants. (DOCX 18 kb)

## Abbreviations

EPR: Electronic patient record
ERS: Electronic record system
GP: General practitioner
HCA: Healthcare assistant
IT: Information technology
NHS: National Health Service
NPfIT: Natioanl Programme for IT in the NHS
NPT: Normalisation process theory
UK: United Kingdom
US: United States
